# Maternal nutritional risk factors for pre-eclampsia incidence: findings from a narrative scoping review

**DOI:** 10.1186/s12978-022-01485-9

**Published:** 2022-09-05

**Authors:** Mai-Lei Woo Kinshella, Shazmeen Omar, Kerri Scherbinsky, Marianne Vidler, Laura A. Magee, Peter von Dadelszen, Sophie E. Moore, Rajavel Elango, The PRECISE Conceptual Framework Working Group, Peter von Dadelszen, Laura A. Magee, Lucilla Poston, Hiten D. Mistry, Marie-Laure Volvert, Cristina Escalona Lopez, Sophie Moore, Rachel Tribe, Andrew Shennan, Tatiana Salisbury, Lucy Chappell, Rachel Craik, Marleen Temmerman, Angela Koech Etyang, Sikolia Wanyonyi, Geoffrey Omuse, Patricia Okiro, Grace Mwashigadi, Esperança Sevene, Helena Boene, Corssino Tchavana, Eusebio Macete, Carla Carillho, Lazaro Quimice, Sonia Maculuve, Donna Russell, Ben Baratt, Joy Lawn, Hannah Blencowe, Veronique Filippi, Matt Silver, Prestige Tatenda Makanga, Liberty Makacha, Yolisa Dube, Newton Nyapwere, Reason Mlambo, Umberto D’Alessandro, Anna Roca, Melisa Martinez-Alvarez, Hawanatu Jah, Brahima Diallo, Abdul Karim Sesay, Fatima Touray, Abdoulie Sillah, Alison Noble, Aris Papageorghiou, Judith Cartwright, Guy Whitley, Sanjeev Krishna, Rosemarie Townsend, Asma Khalil, Marianne Vidler, Joel Singer, Jing Li, Jeffrey Bone, Mai-Lei Woo Kinshella, Kelly Pickerill, Ash Sandhu, Tu Domena, Rajavel Elango, William Stones

**Affiliations:** 1 Department of Obstetrics and Gynaecology, BC Children’s and Women’s Hospital and University of British Columbia, Vancouver, Canada; 2 Department of Pediatrics, Rm170, BC Children’s and Women’s Hospital, University of British Columbia, 950 West 28th Avenue, Vancouver, Canada; 3 Department of Women & Children’s Health, King’s College London, London, UK; 4 MRC Unit The Gambia at the London, School of Hygiene and Tropical Medicine, Fajara, The Gambia; 5 School of Population and Public Health, University of British Columbia, Vancouver, Canada

## Abstract

**Background:**

Pre-eclampsia is a leading cause of maternal mortality and morbidity that involves pregnancy-related stressors on the maternal cardiovascular and metabolic systems. As nutrition is important to support optimal development of the placenta and for the developing fetus, maternal diets may play a role in preventing pre-eclampsia. The purpose of this scoping review is to map the maternal nutritional deficiencies and imbalances associated with pre-eclampsia incidence and discuss evidence consistency and linkages with current understandings of the etiology of pre-eclampsia.

**Methods:**

A narrative scoping review was conducted to provide a descriptive account of available research, summarize research findings and identify gaps in the evidence base. Relevant observational studies and reviews of observational studies were identified in an iterative two-stage process first involving electronic database searches then more sensitive searches as familiarity with the literature increased. Results were considered in terms of their consistency of evidence, effect sizes and biological plausibility.

**Results:**

The review found evidence for associations between nutritional inadequacies and a greater risk of pre-eclampsia. These associations were most likely mediated through oxidative stress, inflammation, maternal endothelial dysfunction and blood pressure in the pathophysiology of pre-eclampsia. Maternal nutritional risk factors for pre-eclampsia incidence with the strongest consistency, effect and biological plausibility include vitamin C and its potential relationship with iron status, vitamin D (both on its own and combined with calcium and magnesium), and healthy dietary patterns featuring high consumption of fruits, vegetables, whole grains, fish, seafood and monounsaturated vegetable oils. Foods high in added sugar, such as sugary drinks, were associated with increased risk of pre-eclampsia incidence.

**Conclusion:**

A growing body of literature highlights the involvement of maternal dietary factors in the development of pre-eclampsia. Our review findings support the need for further investigation into potential interactions between dietary factors and consideration of nutritional homeostasis and healthy dietary patterns. Further research is recommended to explore gestational age, potential non-linear relationships, dietary diversity and social, cultural contexts of food and meals.

**Supplementary Information:**

The online version contains supplementary material available at 10.1186/s12978-022-01485-9.

## Plain language summary

Pre-eclampsia is a condition of high blood pressure during the second half of pregnancy with signs of damage to another organ system, often the liver and kidneys. It is a serious and potentially deadly disease and is the second top cause of deaths related to pregnancy and childbirth globally. Though the exact cause of pre-eclampsia is unclear, researchers have discovered that pre-eclampsia develops through abnormal development of the placenta, which is the interface between the growing baby and the mother in the womb. The placenta helps to transfer nutrients, oxygen and waste between the mother and fetus. Nutrition has important roles to play in the development of the placenta and certain vitamins and minerals have clinical properties that may help prevent pre-eclampsia. We conducted a review to summarize observational studies on maternal nutritional risk factors associated with the development of pre-eclampsia. Promising maternal dietary factors that fit with current understandings of how pre-eclampsia develops include vitamin C and its potential relationship with iron, calcium and vitamin D. Healthy dietary patterns with high consumption of fruits, vegetables, whole grains, fish and seafood and monounsaturated vegetable oils are likely beneficial. Foods high in added sugar, such as sugary drinks, may be linked to higher rates of developing pre-eclampsia. Instead of focusing on single nutrient deficiencies, our findings support a broader approach to explore interrelationships between dietary factors and balanced healthy dietary intake for the prevention of pre-eclampsia.

**Supplementary Information:**

The online version contains supplementary material available at 10.1186/s12978-022-01485-9.

## Introduction

Pre-eclampsia is the most serious of the hypertensive disorders of pregnancy (HDP), which are the second leading direct cause of maternal and infant deaths associated with over 46,000 maternal deaths, 416,000 stillbirths and 1.5–2 million neonatal deaths annually [1, 2]. Pre-eclampsia is characterized by new onset hypertension after 20 weeks gestation with proteinuria and/or signs of organ damage, most commonly the liver and kidneys [2–4]. HDPs complicates 5–10% of pregnancies, including 2–4% with pre-eclampsia, according to global estimates [4, 5]. Pre-eclampsia is estimated to contribute up to a third of all cases of serious maternal morbidity, with approximately 5% of women with pre-eclampsia requiring admission to intensive care, and associated with development of non-communicable diseases such as cardiovascular disease, type II diabetes and renal impairment in the long-term [1, 6]. Pre-eclampsia is closely associated with low birthweight (< 2500 g), documented as a contributing factor in 12% of infants born with intrauterine growth restriction and 19% of preterm birth [6–9].

Although not fully understood, a growing body of evidence finds that pre-eclampsia is a pregnancy-specific inflammatory disorder with two major pathways of pathophysiology [3]. Placental pre-eclampsia, generally developing before 34 weeks gestation, arises from abnormal vascular development of the placenta and inadequate transformation of the maternal spiral arteries to adequately supply nutrients to the placenta and fetus [3, 10]. Maternal pre-eclampsia, generally developing after 34 weeks and postpartum, arises from interactions between a normal placenta and maternal cardiovascular risk factors such as hypertension, renal disease, overweight, and diabetes [3]. Pre-eclampsia of maternal or placental origin are not mutually exclusive and there is an overlap in influencing factors [3]. Certain micronutrients have antioxidant and anti-inflammatory properties, and dietary deficiencies may contribute to poor placentation. Adaptive responses to maternal malnutrition include the regulation of specific nutrient transporters and blood flow, and thus maternal nutrition is hypothesized to be associated with the development of pre-eclampsia [6, 7, 10, 11].

While a proposed role for diet in the origins of pre-eclampsia is well-established [10–12], evidence from nutritional supplementation trials remains elusive [10, 12]. Although micronutrients are good candidates for pre-eclampsia prevention due to their antioxidant, anti-inflammatory or vasoactive proprieties, current evidence does not support routine supplementation of vitamins C, D or E for the prevention or treatment of pre-eclampsia [10]. High-dose calcium (i.e., at least 1 g/day) may reduce the risk of pre-eclampsia in women with low dietary calcium intake [10]. While supplementation in the latter half of pregnancy may reduce high blood pressure, this timing may be too late to influence placental development largely occurring in early gestation [13]. A multi-country placebo-controlled trial evaluating low-dose calcium replacement (500 mg/day) given preconception and in early pregnancy to women with a history of severe pre-eclampsia and low dietary intake (women in both arms receiving open label 1500 mg/day from 20 weeks) observed a marginal effect on the incidence of pre-eclampsia in a subsequent pregnancy amongst women who were compliant with the intervention [14]. However, the efficacy of supplementation is conceptually a different research question than understanding the association between nutritional deficiencies and risk of pre-eclampsia; the first emphasizes modifiability of specific dietary components related to the condition and involves consideration of the intervention implementation process, while the latter focuses on underlying nutritional deficiencies observed in populations. A 2014 review of dietary factors in the prevention of HDP that excluded purely supplement-based studies yielded more promising results [15]. Higher total energy and lower magnesium and calcium intake during pregnancy were associated with HDP incidence, while maternal diets rich in fruits and vegetables may be beneficial in reducing pre-eclampsia [15]. An updated review is needed, particularly a scoping review of observational studies separated from intervention trials evaluated through the lens of consistency in associations and etiology.

Conventional systematic reviews that value randomized controlled trials (RCTs) as the gold standard of study design and a rigorous criteria for evaluating evidence may not always capture the bigger picture in nutritional epidemiology [16]. This is because diet-related diseases may take time to develop and manifest chronic symptoms, RCTs may not be ethically feasible with dietary deficiencies known to have adverse effects on human health, and dietary modification involves behavioural change inadequately detected in trials with insufficient follow-up time [16]. Therefore, in nutritional epidemiology, it is important to triangulate (a) biological plausibility in association with literature related to physiological mechanisms, (b) consistency of the evidence across study designs and populations, and (c) consideration that small effects for individuals may have a large impact on populations [16].

We performed a scoping review according to the methods described by Arksey and O’Malley [17] to address the research question: What is known from the existing literature about the interaction between maternal nutritional deficiencies and risk of developing pre-eclampsia?

## Methodology

The review is reported according to Preferred Reporting Items for Systematic reviews and Meta-Analyses extension for Scoping Reviews (PRISMA-ScR; Additional file 1) [18] and took an pragmatic approach, as recommended for scoping reviews [17, 19]. Relevant studies were identified in an iterative two-stage process conducted from database inception to January 2021. First, electronic database searches were conducted on Medline Ovid, EMBASE, Cumulative Index to Nursing & Allied Health Literature, Web of Science, JBI Database of Systematic Reviews and Implementation Reports, Database of Abstracts of Reviews of Effects and EBM reviews. EBM reviews includes the Cochrane Database of Systematic Reviews, ACP Journal Club, Clinical Evidence, Evidence-based Mental Health, Evidence-based Nursing, Evidence report/Technology assessment. Registers, including PROSPERO, Cochrane Central Register of Controlled Trials, ClinicalTrials.gov and ICTRP Search Portal of the World Health Organization were also scanned. Two reviewers (MWK and SO) independently reviewed titles, abstracts and full texts with reflexive discussion with a third independent reviewer (KS) at each phase to resolve any disagreements. Existing reviews were prioritized where available and studies were considered if they investigated maternal nutrition and diets, if they examined underlying nutritional status and deficiencies and/or discussions of biological plausibility that linked nutritional deficiencies with hypotheses of the etiology of pre-eclampsia. Nutritional supplementation trials raise questions about compliance and adherence to the intervention protocol as well as duration, dosage and timing of intervention delivery, factors that would be more appropriately evaluated in a systematic review. This scoping review seeks more broadly to understand maternal nutritional status and nutritional deficiencies as exposures on the outcome of pre-eclampsia and other hypertensive disorders of pregnancy. Consequently, our scoping review focused on observational studies and reviews of observational studies. In the second stage of the search process, the first reviewer (MWK) reviewed the studies compiled, reviewed reference lists, snowball searched related articles on Google Scholar and PubMed, and consulted with experts in the field. Over the two stages of this process, search terms broadly included pregnancy hypertension, pregnancy-induced hypertension, gestational hypertension, pre-eclampsia, eclampsia, HELLP syndrome, pregnancy complications, micronutrients, vitamins, minerals, macronutrients, nutritional deficiencies and serum levels (Additional file 2). Categories of nutritional deficiencies were explored with more sensitive searches in the expanded second stage (e.g., low calcium levels, serum calcium, calcium deficiency and Mediterranean diet), as- familiarity with the literature increased.

Data were charted by sifting, sorting and extracting material according to key issues and themes [17]. The first reviewer (MWK) completed primary data charting and collated summaries of findings, which were then discussed iteratively with the reviewer team (SO, KS, RE, SM, LM, PvD) to review results, identify gaps and consolidate interpretation in reporting until consensus was achieved. Results were considered in terms of their biological plausibility, consistency of evidence and effect sizes as recommended [16, 20]. Biological plausibility refers to the potential biological role the nutrient has in the pathophysiology of pre-eclampsia. Consistency of evidence in nutritional epidemiology considers the direction of association across populations, study designs and statistical methods, recommended to be at least a majority of the high quality studies [20]. Effect sizes in nutritional epidemiology are frequently smaller at around 0.8–1.2, but these may still have large public health effects at a population level. Strong associations over 4.0 are viewed with caution unless corroborated by other studies [20].

## Results

Results are presented according to the following components in the development of pre-eclampsia where literature found that maternal nutritional inadequacies may contribute: proper placentation, regulation of placental inflammation and immune system, oxidative stress maternal endothelial dysfunction and high blood pressure (Table 1; Additional file 3). Within each section, key themes from the reviewed material are presented.

**Table 1:** Role of maternal dietary components in the development of pre-eclampsia

Pathophysiology of pre-eclampsia		Dietary components and mechanism
Poor placentation	Defective trophoblast invasion leading to reduced utero-placental blood flow and inflammatory response	Vitamin D • Regulates inflammation in maternal uterine natural killer cells, which supports extravillous trophoblast invasion • Regulates angiogenesis through stimulating VEGF
		Vitamin B9 (folate) and B12 • Regulates homocysteine concentrations, which damages endothelial cells and increases trophoblast apoptosis
	Oxidative stress	Vitamin C and E • Important antioxidants that scavenges oxygen radicals in aqueous phase (C) and in cell membranes (E) • Vitamin C involved in uptake of free iron in plasma
		Essential trace elements • Zinc, copper and selenium as components of important antioxidants (superoxide dismutase and glutathione peroxidase) •Free iron and copper in plasma are pro-oxidative transition metals
	Endothelial dysfunction and systemic inflammatory response	Omega 3 long-chain polyunsaturated fatty acids • Regulates oxidative stress, inflammation and lowers triglyceride concentrations by reducing free fatty acid availability, increasing phospholipid synthesis and decreasing triglyceride-synthesizing enzyme activity • Increased concentration of circulating lipids accumulate in endothelial cells, which decreases release of prostacyclin (an endothelium-derived vasodilator) and results in endothelial dysfunction
Maternal syndrome with clinical signs of pre-eclampsia		Healthy dietary patterns • Maternal metabolic or insulin resistance syndromes associated with endothelial dysfunction or sensitivity • Diets characterized by high consumption of fruits, vegetables and whole grains, fish and seafood high in omega-3 fatty acids, monounsaturated vegetable oils, and low in added sugar are protective against cardiovascular diseases and metabolic syndromes
	High blood pressure	Vitamin D and calcium • Low serum calcium triggers increased secretion of parathyroid hormone (PTH), which stimulates release calcium stores from bone reservoirs, opens calcium channels to increase intracellular calcium, which leads to vasoconstriction and high blood pressure • PTH stimulates calcitriol production and calcitriol increases calcium absorption from the intestine • PTH activates the renin–angiotensin–aldosterone signalling pathway, which increases the vascular volume though sodium and water reabsorption

### Poor placentation, immune response and inflammation

Poor placentation is characteristic in the pre-clinical stage of early-onset/placental pre-eclampsia where shallow cytotrophoblast invasion results in defective remodelling of the maternal spiral arteries [3, 10]. The under-perfused placenta creates angiogenic imbalance in the maternal blood stream and activates maternal inflammatory response [4, 10, 13].

Vitamin D is a fat-soluble vitamin with two major physiological forms, ergocalciferol (D2), which is mainly obtained from plant and vegetable sources, and cholecalciferol (D3), which is mainly synthesized in the skin from seven dehydrocholesterol with exposure to UVB radiation from sunlight [21]. Vitamins D2 and D3 are prohormones first hydroxylated in the liver to produce calcidiol (25-hydroxycholecalciferol, 25OHD), which is frequently used as the best stable and circulating biomarker for vitamin D status [22–24]. 25OHD is further hydroxylated in the kidneys to produce calcitriol (1,25 dihydroxycholecalciferol), which is the active form of the hormone used by the body [21]. Calcitriol synthesis has also been demonstrated in decidua and placenta, and calcitriol has important roles in hormonogenesis and overall placental physiology including stimulating endometrial decidualization and synthesis of vital hormones (estradiol, progesterone, human chorionic gonadotrophin, human placental lactogen) in pregnancy [21, 25]. Calcitriol regulates placental immune response and inflammation in maternal uterine natural killer (uNK) cells by inhibiting inflammatory cytokines (TNF-α, IL-6) produced by trophoblast cells to reduce inflammation and produced by decidual cells in the maternal endometrium for immunosuppression as well as promoting the release of antimicrobial peptides (hCTD, HBD2, HBD3) to restrain infection [21]. uNK cells in turn regulate extravillous trophoblast invasion of the decidua and maternal spiral arteries that is characteristic of poor placentation in pre-eclampsia [21, 26, 27]. In addition, in vitro studies have demonstrated a direct pro-invasive effect of calcitriol on the extravillous trophoblast [26]. Calcitriol is also involved in regulating angiogenesis through stimulating the expression of pro-angiogenic signaling protein, VEGF, in vascular smooth muscle cells [28, 29].

Women with pre-eclampsia had significantly lower 25OHD serum concentrations in comparison with normotensive pregnant women [30, 31]. Most meta-analyses on vitamin D insufficiency (25OHD < 75 nmol/L) and deficiency (25OHD < 50 nmol/L) reported significantly increased odds of pre-eclampsia (insufficiency: OR 2.11, 95% CI 1.36–3.27, I^2^ 0%, 5 studies [30]/deficiency: OR 1.27, 95% CI 0.67–2.42, I^2^ 0%, 2 studies [30]; OR 1.54, I^2^ 58%, other information not reported [31]; OR 1.62, 95% CI 1.36–1.94, I2 50%, 21 studies [32]). However, a recent meta-analysis found that associations were not significant under a random effects model (insufficiency: OR 1.26, 95% CI 0.97–1.82, I^2^ 78%, 11 studies/vitamin D deficiency: OR 1.42, 95% CI 0.99–2.04, I^2^ 83%, 11 studies) highlighting high heterogeneity due to differences in study design and gestational age at time of sampling [33]. A European nested case–controlled study in 83 women with pre-eclampsia matched with 319 normotensive controls found that adequate plasma vitamin D concentration in the third trimester was significantly protective while not significant in first trimester alone [34]. The strongest effect was among women with sufficient vitamin D in both first and third trimesters, which was related to 66% lower odds of pre-eclampsia compared with women who had low vitamin D in the same periods (OR 0.34, 95% CI 0.13–0.86, p = 0.02) [34]. This may suggest that while vitamin D is involved in proper placentation, a further role of vitamin D in calcium homeostasis may be for blood pressure regulation in late gestation, which may play a major part in the linkage between its deficiency and increased risk of pre-eclampsia. This is further discussed below.

In addition to its essential roles in nucleotide synthesis, cell growth and proliferation, and DNA methylation during pregnancy [35], folate/folic acid and vitamin B12 are regulators of plasma homocysteine concentrations [10, 36]. Elevated homocysteine concentrations may cause direct damage to endothelial cells and increase cellular destruction (apoptosis) of trophoblast cells leading to poor placentation [36, 37]. A meta-analysis did not find a significant association between maternal folate deficiencies and pre-eclampsia incidence [38]; however, two large studies with over 1000 participants each (China [36], the Netherlands [37]) highlighted that low plasma folate and high homocysteine concentrations were particularly associated with incidence of pre-eclampsia during early pregnancy when measured around 11–13 weeks gestation [36, 37]. Both studies found strong effect sizes comparing women with the lowest folate concentrations to highest concentrations on pre-eclampsia incidence (Q1 vs Q4 OR 2.27, 95% CI 1.56–3.33, p < 0.001 [36]; Q1 vs Q5 adjusted OR 2.10, 95% CI 1.05–4.20, p = 0.04 [37]).

### Oxidative stress

Poor placentation in the pre-clinical stage progresses to the clinical symptoms of the syndrome in the second stage [3, 7, 11]. However, reduced placental perfusion alone is not considered sufficient to explain pre-eclampsia since other conditions such as intrauterine growth restriction and preterm birth are also associated with poor placental development. Increased oxidative stress resulting from an under-perfused placenta has been proposed as the linkage between the two stages of pre-eclampsia [7, 11]. Oxidative stress occurs when there is an accumulation of free radicals (substances with one or more unpaired electrons) that exceeds the capacity of antioxidant defense mechanisms to mitigate against the harmful effects of free radicals, including damage of cells, lipids and proteins [7, 11, 39–41]. An imbalance between the production of free radicals and antioxidants triggers oxidative stress, which contributes to maternal endothelial cell activation, destruction of trophoblast cells and vasoconstriction that are characteristic of pre-eclampsia [7, 39, 41]. An increased rate of destruction (aponecrosis) of syncytiotrophoblast leads to higher concentration of trophoblast debris in maternal circulation, which subsequently increases systemic inflammatory responses [7, 42, 43].

Vitamin C and E are important antioxidants in humans obtained through dietary intake that help protect against oxidative stress [10, 40, 44]. Vitamin C (ascorbic acid, ascorbate) is an essential hydrophilic micronutrient that scavenges oxygen radicals in the aqueous phase, while vitamin E is the major lipid-soluble micronutrient (tocopherols and tocotrienols) that scavenges oxygen radicals within cell membranes [11, 40, 45]. Vitamin C is considered the “linchpin” antioxidant in humans to replenishes vitamin E and is proposed to be the first antioxidant defense mechanism damaged in women with pre-eclampsia as no other antioxidants are reduced prior to vitamin C being depleted [11, 40, 45]. A meta-analysis found that women with pre-eclampsia had significantly lower third trimester concentrations of vitamin C and E in comparison with normotensive pregnancies (vitamin C SMD − 0.56, 95% CI − 0.83 to − 0.28, I^2^ 91%, 29 studies; vitamin E SMD − 0.42, 95% CI − 0.72 to − 0.13, I^2^ 93%, 34 studies) [46]. While the meta-analysis reported high heterogeneity, there was a consistent direction of effect in the high quality studies (studies with sample sizes over 50 participants), especially with vitamin C [46]. Of the six high quality studies, five case–control studies in Spain [47], Nigeria [48], Uganda [49], Peru [50], and United States [44] found that pre-eclampsia was associated with significant reductions of vitamin C and/or E. Both low dietary intake of vitamin C and low plasma vitamin C concentration were associated with over two-fold higher odds of pre-eclampsia incidence in comparison with pregnant women with adequate vitamin C (dietary intake < 85 mg/day OR 2.1, 95% CI 1.1–3.9 [44]; vitamin C concentration < 2.0 × 10^3^ μg/L OR 2.9, 95% CI 1.56–5.44 [49]). A Bangladeshi case–controlled study with 150 pre-eclampsia cases and 174 healthy pregnant controls found significantly lower concentrations of serum vitamin C among cases combined with elevated levels of lipid peroxidation (p < 0.05) [51]. Another recent Canadian nested case–controlled study with 111 pre-eclampsia cases and 441 normotensive controls observed that antioxidants have potentially stronger associations for early-onset (generally placental) rather than late-onset (generally maternal) pre-eclampsia suggesting the role of oxidative stress in the pathophysiology of placental pre-eclampsia [52].

Essential trace elements including zinc, copper and selenium are vital components for antioxidants synthesized in the body. Zinc and copper are essential components for superoxide dismutase (SOD), while selenium is vital for proper functioning of selenoenzymes, such as glutathione peroxidase (GPx) [10, 53–56]. SOD and GPx are both considered “first line” antioxidants and indispensable to the body’s defense against oxygen radicals [57]. Two meta-analyses found that women with pre-eclampsia had significantly lower concentration of serum zinc (SMD − 0.587, 95% CI − 0.963 to − 0.21 I^2^ 88%, 14 studies [58]; SMD − 0.61, 95% CI − 0.74 to − 0.48, I^2^ 88.5%, 3 studies [59]). A third review found evidence supporting an association between low maternal dietary zinc intake and low birthweight infants (10 out of 16 studies) and severe pre-eclampsia (16 out of 33 studies), but not preterm birth and gestational diabetes [53]. However, there were high rates of heterogeneity among three systematic reviews investigating association between maternal zinc concentrations and pre-eclampsia [53, 58, 59], which may be driven by gestational age of measurement [60]. Evidence for selenium was limited; a UK study indicated mixed results [61] and an Iranian study reported a strong effect between women with low selenium concentration and pre-eclampsia compared to women with high concentration (OR 9.14, 95% CI 2.25–37.01) [56]. Such findings from single trials, however, require further corroboration. A meta-analysis found significantly higher serum copper concentrations among women with pre-eclampsia in comparison to normotensive controls (SMD 0.69, 95% CI 0.54–0.8, I^2^ 97%, 12 studies) [54], likely because free copper in the plasma is pro-oxidant [55].

In addition, vitamin C is actively involved in the uptake of free iron in the plasma (non-transferrin bound iron) and is consequently important for iron homeostasis [62]. While the reactive oxygen species are insufficient to initiate cellular damage directly, transitional metal ions (e.g., free iron) in plasma in particular are a necessary co-factor in the production of the highly reactive hydroxyl radicals that may initiate the process of lipid peroxidation and subsequent endothelial cell damage [63–65]. In the current review, iron was a dietary factor with significant heterogeneity across studies in part due to the number of different indicators associated with iron status, including serum iron concentration but also maternal anaemia measured as low hemoglobin concentrations. Two meta-analyses found that women with pre-eclampsia had significantly higher serum iron concentrations (SMD 0.28, 95% CI 0.11–0.44 [66]; SMD 1.27, 95% CI 0.76–1.78 [67]). Another meta-analysis found a significant association between maternal anaemia (hemoglobin < 11 g/dL) and pre-eclampsia incidence in the first (OR 1.55, 95% CI 1.15–2.10) but not in the second or third trimesters [68]. Another meta-analysis found a dose–response with both low maternal hemoglobin (< 110 g/L) and high maternal hemoglobin (> 130 g/L) significantly associated with higher PE incidence (low Hb OR 1.84, 95% CI 1.31–2.59, high Hb OR 1.48, 95% CI 1.10–2.01) [69].

### Maternal endothelial dysfunction

The stressed placenta triggers endothelial dysfunction and increases systemic inflammatory responses [70]. Dietary components may also contribute. Increased concentrations of circulating lipids may accumulate within endothelial cells, which triggers a decreased release of prostaglandins, specifically prostacyclin [71–73]. Prostacyclin is an effective vasodilator and inhibits platelet aggregation. Reduced prostacyclin levels results in endothelial dysfunction with exacerbated oxidative stress and maternal inflammation [72–74]. While maternal metabolism changes during normal uncomplicated pregnancies, especially in late gestation to meet the needs of the growing fetus [73–76], women with pre-eclampsia a tend to experience greater changes in lipid metabolism. Three meta-analyses [72, 76, 77] found that women with pre-eclampsia have consistently higher serum triglycerides concentrations, compared with normotensive pregnant controls, even after adjusting for maternal BMI [72]. Triglycerides were significantly elevated in first and second trimesters before onset of pre-eclampsia in women who developed the condition [72, 76]. Long chain polyunsaturated fatty acids (PUFA) are significantly associated with lower triglyceride concentrations [78]. For example, omega-3 fatty acids can help reduce triglyceride concentration by reducing free fatty acid availability, increasing phospholipid synthesis and decreasing triglyceride-synthesizing enzyme activity [79]. Omega-3 fatty acids must be obtained from the diet, mainly from fish and marine sources or as α-linolenic acid from vegetable oils [80]. However, the evidence on the association between omega-3 dietary intake and lower risk of pregnancy hypertension from observational studies is inconsistent, with studies in populations with traditionally higher omega-3 intakes reporting significant associations [81–83], but not among populations with low intakes [84, 85]. An observational study from Iceland also cautioned that supplementation alongside traditional fish and cod-liver oil consumption may result in overly high intake of omega-3, with increased the risk of developing HDPs [86].

Pregnancy places stress on maternal cardiovascular and metabolic systems and there is a decrease in insulin sensitivity during gestation to allow the maternal metabolism to meet fetal demands for glucose [87, 88]. Pregnancy may be considered a metabolic and vascular “stress test” [7]. Maternal metabolic or insulin resistance syndromes such as chronic hypertension, dyslipidemia, insulin resistance, diabetes, adiposity, and hemostatic disturbances are associated with endothelial cell dysfunction, which increases sensitivity to placental factors and increases the risk of developing pre-eclampsia [89]. A case–controlled study with 259 women with pre-eclampsia and 297 normotensive controls in the United States found that women with a higher metabolic score based on the World Health Organization (WHO) and the National Cholesterol Education Program (NCEP-ATP III) diagnostic criteria was independently associated with developing pre-eclampsia [90]. Healthy dietary patterns emphasizing high consumption of fruits, vegetables, whole grains, and limited consumption of saturated fats, sodium, red meat, and sweets, such as the Mediterranean, Nordic and Dietary Approaches to Stop Hypertension (DASH) diets, have been shown to be protective against cardiovascular diseases and metabolic syndromes [91–96]. Olive oil is traditional to the Mediterranean region and a staple component of the Mediterranean diet, while the Nordic diet emphasizes locally sourced foods in modern-day Scandinavia and includes rapeseed (canola) oil [95]. Mediterranean and Nordic diets are recommended by the WHO for their protective benefits against cardiovascular diseases and type 2 diabetes [95].

Cohort studies in Greece [97], Netherlands [98] and Australia [99] found that high adherence to a Mediterranean type diet was protective against HDPs. The Generation R cohort study with 3187 pregnant women found that low adherence to a Mediterranean dietary pattern was associated with higher systolic and diastolic blood pressure in pregnancy, though not significantly associated with gestational hypertension or pre-eclampsia incidence [98]. A 9 year follow-up of 3582 women in the Australian Longitudinal Study on Women’s Health found that high adherence to a Mediterranean dietary pattern was significantly associated 42% reduced risk (RR 0.58, 95% CI 0.42–0.81) of developing HDPs in comparison with those with low adherence [99]. The association may be stronger among high-risk women. A cohort of 82 women with early preterm singleton births (≤ 34 weeks) found that low adherence to a Mediterranean diet was associated with an almost four-fold increased odds (OR 3.8, 95% CI 1.3–11.4) of developing gestational hypertension or pre-eclampsia [97]. High adherence to the Nordic diet was significantly associated with 14% reduced odds of any pre-eclampsia (OR 0.86, 95% CI 0.78–0.95) and 29% reduced odds of early onset pre-eclampsia (OR 0.71; 95% CI 0.52–0.96) in comparison to low adherence among the 72,072 women from the Norwegian Mother and Child Cohort Study (MoBa) [100]. In contrast, two large cohort studies from the United States (Project Viva) and Denmark (Danish National Birth Cohort) did not find an association between the DASH diet and pre-eclampsia incidence [101, 102].

The protective benefit of Mediterranean and Nordic diets, which both feature low intake of foods with added sugar, corroborates with findings from two Norwegian cohort studies that reported associations with high consumption of sugary drinks with increased risk of pre-eclampsia incidence [89, 103]. Women who drank 125 ml or more a day of sugar-sweetened carbonated and non-carbonated beverages had 27% higher odds of developing pre-eclampsia (adjusted OR 1.27, 95% CI 1.05–1.54), in comparison to women with no intake after controlling for potential confounders [103]. However, high intake of fruit, though high in natural sugars, was protective against pre-eclampsia [103].

### High blood pressure

Vitamin D supports calcium homeostasis. Concentrations of maternal plasma calcitriol, a vitamin D metabolite, almost double by the end of the third trimester as calcium absorption and placental calcium transport needs intensify for adequate fetal bone mineralization [10, 21]. Calcium was identified early as a potential nutrients in relation to pre-eclampsia as Belizan and Villar observed an inverse relationship between low calcium intake with higher rate of eclampsia incidence in the 1980s [104]. They observed low pre-eclampsia and eclampsia rates among groups in low- and middle-income countries (LMICs) with traditional diets rich in calcium, such as among populations in Ethiopia and among Mayan Indians in Guatemala who traditionally soak their corn in lime prior to cooking and suggested that the association could not be wholly driven by socio-economic factors [104]. Calcium plays an important role in muscle contraction and regulation of water balance in cells in the regulation of blood pressure [10, 105, 106]. Low serum calcium concentrations trigger increases in the secretion of parathyroid hormone (PTH) by the parathyroid gland, which stimulates the release of calcium stores in bone reservoirs into the bloodstream and increases intracellular calcium in vascular smooth muscle cells by opening calcium channels [106–112]. Increases in intracellular calcium can directly contribute to vasoconstriction and subsequent high blood pressure. PTH also stimulates the production of 1alpha-hydroxylase, which catalyzes the synthesis of calcitriol that increases calcium absorption from the intestine [108, 112]. PTH indirectly contributes to high blood volume by activation of the renin–angiotensin–aldosterone signalling pathway, increasing vascular volume though sodium and water reabsorption [106, 107, 112]. Calcitriol decreases renin activity and PTH secretion through negative feedback [112, 113]. Adequate serum calcium concentrations serve as a negative-feedback loop signalling decreased secretion of PTH, stabilizes vascular cell membranes and reduces vasoconstriction [108, 110, 112].

Perhaps because calcium was identified early in the prevention of pre-eclampsia and supplementation trials commenced early, there is less attention to epidemiological studies investigating the association between calcium deficiencies and pre-eclampsia incidence. In contrast to numerous systematic reviews of observational studies with vitamin D on the topic, there was one meta-analysis found for calcium, which found a significant protective effect with an OR of 0.63 (95% CI 0.41–0.97, I^2^ 0%, 7 studies) for high calcium intake during pregnancy (> 1600 mg/day) in comparison with insufficient intake (< 1000 mg/day) on gestational hypertension [15]. While the meta-analysis noted a consistent direction of higher calcium intake as protective [15], two subsequent cross-sectional studies from Haryana, India did not find a significant association between pre-eclampsia and hypocalcemia (serum calcium < 9 mg/day) [114, 115]. However, the authors noted that while the majority of pregnant women had inadequate dietary calcium intakes, dietary intakes did not correspond with serum calcium concentration. This was perhaps due to higher sun exposure among the rural women in the study as vitamin D formation may increase calcium serum concentration [114, 115].

Instead of investigating calcium concentrations in isolation, epidemiological studies more frequently examined calcium and magnesium concentrations together. Magnesium is a calcium channel blocker that reduces intracellular calcium and thus results in vasodilation and reduced blood pressure [110, 116, 117]. While a meta-analysis found that women with HDP had significantly lower concentrations of magnesium (− 8.0, 95% CI − 13.99 to − 1.38, I^2^ 0%, 7 studies) [15], the consistency of evidence was mixed. There was higher consistency in case–controlled studies with more than 100 participants [118–121] and less consistency in case–controlled studies with 100 participants or less [110, 116, 117, 122, 123].

## Discussion

The purpose of this narrative scoping review was to map current evidence of maternal nutritional deficiencies and imbalances associated with the development of pre-eclampsia from observational studies and reviews of observational studies. There were a number of promising maternal dietary factors associated with risk of pre-eclampsia incidence that aligned with current understandings of biological mechanisms, including vitamin C as an essential antioxidant and its relationship with iron status, and vitamin D for its roles in placental development and together with calcium for blood pressure regulation. While evidence on omega-3 long-chain PUFAs seemed inconsistent, diets protective against cardiovascular and metabolic syndromes were more promising, particularly when characterized by high consumption of fruits, vegetables and whole grains as well as fish and seafood high in omega-3 fatty acids and monounsaturated vegetable oils. Associations between added sugar intake and pre-eclampsia incidence may also be an area to further explore as evidence is limited, though reported associations are strong. In addition to added sugar, other current gaps include limited literature regarding B vitamins, zinc and other essential trace elements, dietary diversity and recent studies on dietary calcium.

With the exception of calcium, where decades of intervention trials corroborate with early epidemiological observations and understandings of biological mechanisms on evidence of a protective effect against pre-eclampsia, many potential maternal dietary factors are not supported by results of supplementation trials [124]. For example, though maternal serum vitamin C [52], zinc [58] and iron [67] have strong associations with the development of pre-eclampsia and make biological sense, supplementation resulted in non-significant effects [124–127]. Findings from this scoping review may help to shed light on the discrepancy. First, the relationship between dietary factors and maternal conditions may not be linear for certain nutrients. This was most clearly described with iron as both high and low hemoglobin levels were associated with higher risk of developing pre-eclampsia [69] but also hinted with omega-3 fatty acids [86]. Consequently, heterogeneity of results may in part be due a focus on nutritional deficiencies rather than also when concentrations are too high. Secondly, gestational age is an important modifying factor because dietary components may be involved in different processes and demands over the course of the pregnancy. Studies with iron and vitamin D demonstrate that effects may be time-dependent [34, 68]. Periconceptual nutrition from 14 weeks before conception to 10–12 weeks gestation when women may not be aware of their pregnancy status may be particularly important for placentation [128–130], though there remains a gap on understanding dietary components for that phase of disease development [11]. Furthermore, interrelationships between dietary factors complicate the direct linkage between dietary intake and its metabolism. For example, the Indian studies did not find a correlation between dietary intake and serum calcium concentration perhaps due to unreliable methods in accessing dietary intake or because their rural study population had more exposure to sunlight and subsequent synthesis of vitamin D [114, 115]. Potential synergies of dietary factors, such as vitamin C and iron for oxidative stress and vitamin D, calcium and magnesium for regulation of blood pressure, may shed light onto why single supplement trials may not have been as effective as desired in pre-eclampsia prevention. Further research is recommended to investigate gestational age, potential non-linear relationships and interactions between dietary factors.

A broader approach to explore interrelationships between dietary factors and considering balanced dietary intake supports the potential for healthy dietary patterns in the prevention of pre-eclampsia, as previously suggested and further strengthened in this review. The review by Schoenaker et al. found a non-significant trend towards the protective effect of healthy dietary patterns high in fruits and vegetables [15]. Since the 2014 Schoenaker et al. review, there has been further evidence in cohort studies of the protective effect of Mediterranean and Nordic diets on lowering risk of pre-eclampsia incidence [97, 99, 100]. However, two recent large cohort studies did not find a significant association between the DASH diet and risk of pre-eclampsia incidence [101, 102]. This suggests that healthy diets comprise of more than high intake of fruits, vegetables and whole grains, which the three diets have in common. The DASH diet aims to be low in total fat while Mediterranean and Nordic diets include fish and seafood (high in omega-3 long-chain PUFAs) as well as olive oil in the Mediterranean diet and rapeseed (canola) oil in the Nordic diet (both monounsaturated fats). Unlike the DASH diet, the Mediterranean and Nordic diets also emphasizes the cultural significance of these diets, including their linkages to identity, local food systems, and sociality of shared meals together with family and friends [95]. Further research into the social and cultural contexts of meals and eating may complement our understandings of maternal dietary patterns and their associated maternal health risks. Additionally, more research is needed to understand food groups and their combinations in association with pre-eclampsia incidence rates, particularly in LMICs. Cohort studies examining maternal dietary patterns are largely conducted in high-income countries and less is known in resource-limited settings.

In contrast to other systematic reviews, the focus of this review is less on evaluation of effect sizes. A limitation is that this is not a systematic review and does not provide evidence towards clinical practice. Rather, the emphasis of this scoping review was to map the landscape of the epidemiological evidence. Significant results within diverse populations was emphasized as a strength. A strength of this review is its extensive and iterative search strategy that adapted to knowledge gained in the process.

## Conclusion

The review finds a growing body of literature describing the associations between nutritional inadequacies and higher risk of pre-eclampsia potentially through oxidative stress, inflammation, maternal endothelial dysfunction and blood pressure in the pathophysiology of pre-eclampsia. On the one hand, these findings confirm our understandings of oxidative stress, inflammation, maternal endothelial dysfunction and blood pressure in the development of pre-eclampsia while on the other hand, reinforce the potential role of maternal dietary factors. Rather than examining individual macro- or micronutrients in isolation, findings from the review suggests looking broader to interactions between dietary factors, and shifting from a narrow focus on nutritional deficiencies to wider discussions of nutritional homeostasis and healthy dietary patterns to reduce risk of HDPs.

## Supplementary Information

**Additional file 1.** Preferred Reporting Items for Systematic reviews and Meta-Analyses extension for Scoping Reviews (PRISMA-ScR) Checklist.

**Additional file 2.** Search terms.

**Additional file 3.** PRISMA flow diagram.

## Data Availability

All data generated or analysed during this study are included in this published article.

## Abbreviations

DASH: Dietary Approaches to Stop Hypertension
HDP: Hypertensive disorders of pregnancy
LMICs: Low- and middle-income countries
PRISMA-ScR: Preferred Reporting Items for Systematic reviews and Meta-Analyses extension for Scoping Reviews
PTH: Parathyroid hormone
PUFA: Polyunsaturated fatty acids
RCT: Randomized controlled trials
uNK: Uterine natural killer cells
WHO: World Health Organization

## References

[1] von Dadelszen P, Magee LA (2016). Preventing deaths due to the hypertensive disorders of pregnancy. Best Pract Res Clin Obstet Gynaecol.

[2] Brown MA, Magee LA, Kenny LC, Karumanchi SA, McCarthy FP, Saito S (2018). Hypertensive disorders of pregnancy: ISSHP classification, diagnosis, and management recommendations for international practice. Hypertension.

[3] von Dadelszen P, Ayres de Campos D, Barivalala W, Magee L, von Dadelszen P, Stones W, Mathai M (2016). Classification of the hypertensive disorders of pregnancy. The FIGO textbook of pregnancy hypertension.

[4] Magee LA, Sharma S, Nathan HL, Adetoro OO, Bellad MB, Goudar S (2019). The incidence of pregnancy hypertension in India, Pakistan, Mozambique, and Nigeria: a prospective population-level analysis. PLoS Med.

[5] Payne B, Hanson C, Sharma S, Magee L, von Dadelszen P, Magee L, von Dadelszen P, Stones W, Mathai M (2016). Epidemiology of the hypertensive disorders of pregnancy. The FIGO textbook of pregnancy hypertension.

[6] Dodd JM, O’Brien C, Grivell RM (2014). Preventing pre-eclampsia—are dietary factors the key?. BMC Med.

[7] Xu H, Shatenstein B, Luo Z-C, Wei S, Fraser W (2009). Role of nutrition in the risk of preeclampsia. Nutr Rev.

[8] Hewitt BG, Newnham JP (1988). A review of the obstetric and medical complications leading to the delivery of infants of very low birthweight. Med J Aust.

[9] Henderson JJ, McWilliam OA, Newnham JP, Pennell CE (2012). Preterm birth aetiology 2004–2008. Maternal factors associated with three phenotypes: spontaneous preterm labour, preterm pre-labour rupture of membranes and medically indicated preterm birth. J Matern Neonatal Med.

[10] Achamrah N, Ditisheim A (2018). Nutritional approach to preeclampsia prevention. Curr Opin Clin Nutr Metabolic Care.

[11] Roberts JM, Balk JL, Bodnar LM, Belizá JM, Bergel E, Martinez A (2003). Nutrient involvement in preeclampsia. J Nutr.

[12] Sibai BM (1998). Prevention of preeclampsia: a big disappointment. Am J Obstet Gynecol.

[13] Hofmeyr GJ, Lawrie TA, Atallah ÁN, Duley L, Torloni MR (2018). Calcium supplementation during pregnancy for preventing hypertensive disorders and related problems. Cochrane Database Syst Rev.

[14] Hofmeyr GJ, Betrán AP, Singata-Madliki M, Cormick G, Munjanja SP, Fawcus SS (2019). Prepregnancy and early pregnancy calcium supplementation among women at high risk of pre-eclampsia: a multicentre, double-blind, randomised, placebo-controlled trial. Lancet.

[15] Schoenaker DAJM, Soedamah-Muthu SS, Mishra GD (2014). The association between dietary factors and gestational hypertension and pre-eclampsia: a systematic review and meta-analysis of observational studies. BMC Med.

[16] Neuhouser ML (2020). Red and processed meat: more with less?. Am J Clin Nutr.

[17] Arksey H, O’Malley L (2005). Scoping studies: towards a methodological framework. Int J Soc Res Methodol Theory Pract.

[18] Tricco AC, Lillie E, Zarin W, O’Brien KK, Colquhoun H, Levac D (2018). PRISMA Extension for Scoping Reviews (PRISMA-ScR): checklist and explanation. Ann Intern Med.

[19] Munn Z, Peters MDJ, Stern C, Tufanaru C, McArthur A, Aromataris E (2018). Systematic review or scoping review? Guidance for authors when choosing between a systematic or scoping review approach. BMC Med Res Methodol.

[20] Potischman N, Weed DL (1999). Causal criteria in nutritional epidemiology. Am J Clin Nutr.

[21] Olmos-Ortiz A, Avila E, Durand-Carbajal M, Díaz L (2015). Regulation of calcitriol biosynthesis and activity: focus on gestational vitamin D deficiency and adverse pregnancy outcomes. Nutrients.

[22] Brannon PM, Picciano MF (2011). Vitamin D in pregnancy and lactation in humans. Annu Rev Nutr.

[23] Agarwal S, Kovilam O, Agrawal DK (2018). Vitamin D and its impact on maternal-fetal outcomes in pregnancy: a critical review. Crit Rev Food Sci Nutr.

[24] Kaludjerovic J, Vieth R (2010). Relationship between vitamin D during perinatal development and health. J Midwifery Womens Health.

[25] Barrera D, Díaz L, Noyola-Martínez N, Halhali A (2015). Vitamin D and inflammatory cytokines in healthy and preeclamptic pregnancies. Nutrients.

[26] Chan SY, Susarla R, Canovas D, Vasilopoulou E, Ohizua O, McCabe CJ (2015). Vitamin D promotes human extravillous trophoblast invasion in vitro. Placenta.

[27] Gaynor LM, Colucci F (2017). Uterine natural killer cells: Functional distinctions and influence on pregnancy in humans and mice. Front Immunol.

[28] Hyppönen E, Cavadino A, Williams D, Fraser A, Vereczkey A, Fraser WD (2014). Vitamin D and pre-eclampsia: original data, systematic review and meta-analysis. Ann Nutr Metab.

[29] Cardus A, Panizo S, Encinas M, Dolcet X, Gallego C, Aldea M (2009). 1,25-Dihydroxyvitamin D3 regulates VEGF production through a vitamin D response element in the VEGF promoter. Atherosclerosis.

[30] Aghajafari F, Nagulesapillai T, Ronksley PE, Tough SC, O’Beirne M, Rabi DM (2013). Association between maternal serum 25-hydroxyvitamin D level and pregnancy and neonatal outcomes: systematic review and meta-analysis of observational Studies. BMJ.

[31] Akbari S, Khodadadi B, Ahmadi SAY, Abbaszadeh S, Shahsavar F (2018). Association of vitamin D level and vitamin D deficiency with risk of preeclampsia: a systematic review and updated meta-analysis. Taiwan J Obstet Gynecol.

[32] Yuan Y, Tai W, Xu P, Fu Z, Wang X, Long W (2019). Association of maternal serum 25-hydroxyvitamin D concentrations with risk of preeclampsia: a nested case-control study and meta-analysis. J Matern Neonatal Med.

[33] Aguilar-Cordero MJ, Lasserrot-Cuadrado A, Mur-Villar N, León-Ríos XA, Rivero-Blanco T, Pérez-Castillo IM (2020). Vitamin D, preeclampsia and prematurity: a systematic review and meta-analysis of observational and interventional studies. Midwifery.

[34] Benachi A, Baptiste A, Taieb J, Tsatsaris V, Guibourdenche J, Senat MV (2020). Relationship between vitamin D status in pregnancy and the risk for preeclampsia: a nested case-control study. Clin Nutr.

[35] Pisal H, Dangat K, Randhir K, Khaire A, Mehendale S, Joshi S (2019). Higher maternal plasma folate, vitamin B12 and homocysteine levels in women with preeclampsia. J Hum Hypertens.

[36] Liu C, Luo D, Wang Q, Ma Y, Ping L, Wu T (2020). Serum homocysteine and folate concentrations in early pregnancy and subsequent events of adverse pregnancy outcome: the Sichuan Homocysteine study. BMC Pregnancy Childbirth.

[37] Bergen NE, Jaddoe VWV, Timmermans S, Hofman A, Lindemans J, Russcher H (2012). Homocysteine and folate concentrations in early pregnancy and the risk of adverse pregnancy outcomes: the generation R study. BJOG Int J Obstet Gynaecol.

[38] Ray JG, Laskin CA (1999). Folic acid and homocyst(e)ine metabolic defects and the risk of placental abruption, pre-eclampsia and spontaneous pregnancy loss: a systematic review. Placenta.

[39] Poston L, Raijmakers MTM (2004). Trophoblast oxidative stress, antioxidants and pregnancy outcome—a review. Placenta.

[40] Rumbold AR, Maats FHE, Crowther CA (2005). Dietary intake of vitamin C and vitamin E and the development of hypertensive disorders of pregnancy. Eur J Obstet Gynecol Reprod Biol.

[41] Bilodeau JF, Hubel CA (2003). Current concepts in the use of antioxidants for the treatment of preeclampsia. J Obstet Gynaecol Can.

[42] Redman CWG, Sargent IL (2000). Placental debris, oxidative stress and pre-eclampsia. Placenta.

[43] Hung TH, Skepper JN, Charnock-Jones DS, Burton GJ (2002). Hypoxia-reoxygenation: a potent inducer of apoptotic changes in the human placenta and possible etiological factor in preeclampsia. Circ Res.

[44] Zhang C, Williams MA, King IB, Dashow EE, Sorensen TK, Frederick IO (2002). Vitamin C and the risk of preeclampsia: results from dietary questionnaire and plasma assay. Epidemiology.

[45] Niki E (1987). Interaction of ascorbate and α-tocopherol. Ann N Y Acad Sci.

[46] Cohen JM, Beddaoui M, Kramer MS, Platt RW, Basso O, Kahn SR (2015). Maternal antioxidant levels in pregnancy and risk of preeclampsia and small for gestational age birth: a systematic review and meta-analysis. PLoS ONE.

[47] Gratacós E, Casals E, Deulofeu R, Gómez O, Cararach V, Alonso PL (1999). Serum and placental lipid peroxides in chronic hypertension during pregnancy with and without superimposed preeclampsia. Hypertens Pregnancy.

[48] Ikpen MA, Eigbefoh J, Eifediyi RA, Isabu PA, Okogbenin S, Okogbo FO (2012). Determination of antioxidant status of pre-eclamptic and normotensive sub-rural Nigerian pregnant women at the Irrua specialist teaching hospital, Irrua, Edo State. J Matern Neonatal Med.

[49] Kiondo P, Welishe G, Wandabwa J, Wamuyu-Maina G, Bimenya GS, Okong P (2012). Plasma Vitamin C concentration in pregnant women with preeclampsia in Mulago hospital, Kampala, Uganda. Afr Health Sci.

[50] Zhang C, Williams MA, Sanchez SE, King IB, Ware-Jauregui S, Larrabure G (2001). Plasma concentrations of carotenoids, retinol, and tocopherols in preeclamptic and normotensive pregnant women. Am J Epidemiol.

[51] Shahid Sarwar M, Chandra Sarkar R, Bhowmick R, Masudur S, Dewan R, Ahmed MU (2015). Effect of socio-economic status and estimation of lipid peroxidation and antioxidant in preeclamptic pregnant women: a case-control study. Hypertens Pregnancy.

[52] Cohen JM, Kramer MS, Platt RW, Basso O, Evans RW, Kahn SR (2015). The association between maternal antioxidant levels in midpregnancy and preeclampsia. Am J Obstet Gynecol.

[53] Wilson R, Grieger J, Bianco-Miotto T, Roberts C (2016). Association between maternal zinc status, dietary zinc intake and pregnancy complications: a systematic review. Nutrients.

[54] Fan Y, Kang Y, Zhang M (2016). A meta-analysis of copper level and risk of preeclampsia: evidence from 12 publications. Biosci Rep.

[55] Fenzl V, Flegar-Meštrić Z, Perkov S, Andrišić L, Tatzber F, Žarković N (2013). Trace elements and oxidative stress in hypertensive disorders of pregnancy. Arch Gynecol Obstet.

[56] Ghaemi SZ, Forouhari S, Dabbaghmanesh MH, Sayadi M, Bakhshayeshkaram M, Vaziri F (2013). A prospective study of selenium concentration and risk of preeclampsia in pregnant Iranian women: a nested case-control study. Biol Trace Elem Res.

[57] Ighodaro OM, Akinloye OA (2018). First line defence antioxidants-superoxide dismutase (SOD), catalase (CAT) and glutathione peroxidase (GPX): their fundamental role in the entire antioxidant defence grid. Alex J Med.

[58] Ma Y, Shen X, Zhang D (2015). The relationship between serum zinc level and preeclampsia: a meta-analysis. Nutrients.

[59] Zhu Q, Zhang L, Chen X, Zhou J, Liu J, Chen J (2016). Association between zinc level and the risk of preeclampsia: a meta-analysis. Arch Gynecol Obstet.

[60] Jain S, Sharma P, Kulshreshtha S, Mohan G, Singh S (2010). The role of calcium, magnesium, and zinc in pre-eclampsia. Biol Trace Elem Res.

[61] Rayman MP, Bath SC, Westaway J, Williams P, Mao J, Vanderlelie JJ (2015). Selenium status in UK pregnant women and its relationship with hypertensive conditions of pregnancy. Br J Nutr.

[62] Lane DJR, Richardson DR (2014). The active role of vitamin C in mammalian iron metabolism: much more than just enhanced iron absorption!. Free Radic Biol Med.

[63] Rayman MP, Barlis J, Evans RW, Redman CWG, King LJ (2002). Abnormal iron parameters in the pregnancy syndrome preeclampsia. Am J Obstet Gynecol.

[64] Taheripanah R, Farkush P (2007). Relation between serum ferritin and iron parameters with preeclampsia. J Fam Reprod Health.

[65] Mannaerts D, Faes E, Cos P, Briedé JJ, Gyselaers W, Cornette J (2018). Oxidative stress in healthy pregnancy and preeclampsia is linked to chronic inflammation, iron status and vascular function. PLoS ONE.

[66] Liu JX, Chen D, Li MX, Hua Y (2019). Increased serum iron levels in pregnant women with preeclampsia: a meta-analysis of observational studies. J Obstet Gynaecol (Lahore).

[67] Song QY, Luo WP, Zhang CX (2015). High serum iron level is associated with an increased risk of hypertensive disorders during pregnancy: a meta-analysis of observational studies. Nutr Res.

[68] Jung J, Rahman MM, Rahman MS, Swe KT, Islam MR, Rahman MO (2019). Effects of hemoglobin levels during pregnancy on adverse maternal and infant outcomes: a systematic review and meta-analysis. Ann N Y Acad Sci.

[69] Young MF, Oaks B, Tandon S, Martorell R, Dewey K, Wendt A. Maternal hemoglobin concentrations across pregnancy and maternal and child health: a systematic review and meta-analysis (P11-033-19). Curr Dev Nutr. 2019;3(Supplement_1). 10.1111/nyas.14093 PMC6767572 30994929

[70] Burton GJ, Redman CW, Roberts JM, Moffett A (2019). Pre-eclampsia: pathophysiology and clinical implications. BMJ.

[71] Smith JB (1981). Prostaglandins and platelet aggregation. Acta Med Scand.

[72] Spracklen CN, Smith CJ, Saftlas AF, Robinson JG, Ryckman KK (2014). Systematic reviews and meta-and pooled analyses maternal hyperlipidemia and the risk of preeclampsia: a meta-analysis. Am J Epidemiol.

[73] Wojcik-Baszko D, Charkiewicz K, Laudanski P (2018). Role of dyslipidemia in preeclampsia—a review of lipidomic analysis of blood, placenta, syncytiotrophoblast microvesicles and umbilical cord artery from women with preeclampsia. Prostaglandins Other Lipid Mediat.

[74] Sattar N, Greer IA (1999). Lipids and the pathogenesis of pre-eclampsia. Curr Obstet Gynaecol.

[75] Barrett HL, Nitert D, David Mcintyre H, Callaway LK (2014). Maternal lipids in pre-eclampsia: innocent bystander or culprit?. Hypertens Pregnancy.

[76] Gallos I, Sivakumar K, Kilby M, Coomarasamy A, Thangaratinam S, Vatish M (2013). Pre-eclampsia is associated with, and preceded by, hypertriglyceridaemia: a meta-analysis. BJOG Int J Obstet Gynaecol.

[77] Ray J, Diamond P, Singh G, Bell C (2006). Brief overview of maternal triglycerides as a risk factor for pre-eclampsia. BJOG Int J Obstet Gynaecol.

[78] Grimsgaard S, Bønaa KH, Bjerve KS (2000). Fatty acid chain length and degree of unsaturation are inversely associated with serum triglycerides. Lipids.

[79] Harris WS, Bulchandani D (2006). Why do omega-3 fatty acids lower serum triglycerides?. Curr Opin Lipidol.

[80] Muskiet FAJ, van Goor SA, Kuipers RS, Velzing-Aarts FV, Smit EN, Bouwstra H (2006). Long-chain polyunsaturated fatty acids in maternal and infant nutrition. Prostaglandins Leukot Essent Fat Acids.

[81] Kesmodel U (1997). Marine n-3 fatty acid and calcium intake in relation to pregnancy induced hypertension, intrauterine growth retardation, and preterm delivery a case-control study. Acta Obstet Gynecol Scand.

[82] Popeski D, Ebbeling LR, Brown PB, Hornstra G, Gerrard JM (1991). Blood pressure during pregnancy in Canadian Inuit: community differences related to diet. Can Med Assoc J.

[83] Arvizu M, Afeiche MC, Hansen S, Halldorsson TF, Olsen SF, Chavarro JE (2019). Fat intake during pregnancy and risk of preeclampsia: a prospective cohort study in Denmark. Eur J Clin Nutr.

[84] Mahomed K, Williams MA, King IB, Mudzamiri S, Woelk GB (2007). Erythrocyte OMEGA-3, OMEGA-6 and trans fatty acids in relation to risk of preeclampsia among women delivering at Harare Maternity Hospital, Zimbabwe. Physiol Res.

[85] Oken E, Ning Y, Rifas-Shiman SL, Rich-Edwards JW, Olsen SF, Gillman MW (2007). Diet during pregnancy and risk of preeclampsia or gestational hypertension. Ann Epidemiol.

[86] Olafsdottir A, Skuladottir G, Thorsdottir I, Hauksson A, Thorgeirsdottir H, Steingrimsdottir L (2006). Relationship between high consumption of marine fatty acids in early pregnancy and hypertensive disorders in pregnancy. BJOG Int J Obstet Gynaecol.

[87] Salzer L, Tenenbaum-Gavish K, Hod M (2015). Metabolic disorder of pregnancy (understanding pathophysiology of diabetes and preeclampsia). Best Pract Res Clin Obstet Gynaecol.

[88] Kaaja RJ, Greer IA (2005). Manifestations of chronic disease during pregnancy. J Am Med Assoc.

[89] Clausen T, Slott M, Solvoll K, Drevon CA, Vollset SE, Henriksen T (2001). High intake of energy, sucrose, and polyunsaturated fatty acids is associated with increased risk of preeclampsia. Am J Obstet Gynecol.

[90] Mazar RM, Srinivas SK, Sammel MD, Andrela CM, Elovitz MA (2007). Metabolic score as a novel approach to assessing preeclampsia risk. Am J Obstet Gynecol.

[91] Kastorini CM, Milionis HJ, Esposito K, Giugliano D, Goudevenos JA, Panagiotakos DB (2011). The effect of mediterranean diet on metabolic syndrome and its components: a meta-analysis of 50 studies and 534,906 individuals. J Am Coll Cardiol.

[92] Esposito K, Kastorini CM, Panagiotakos DB, Giugliano D (2013). Mediterranean diet and metabolic syndrome: an updated systematic review. Rev Endocr Metab Disord.

[93] Ramezani-Jolfaie N, Mohammadi M, Salehi-Abargouei A (2019). The effect of healthy Nordic diet on cardio-metabolic markers: a systematic review and meta-analysis of randomized controlled clinical trials. Eur J Nutr.

[94] Salehi-Abargouei A, Maghsoudi Z, Shirani F, Azadbakht L (2013). Effects of Dietary Approaches to Stop Hypertension (DASH)-style diet on fatal or nonfatal cardiovascular diseases-Incidence: a systematic review and meta-analysis on observational prospective studies. Nutrition.

[95] Renzella J, Townsend N, Jewell J, Breda J, Roberts N, Rayner M, et al. What national and subnational interventions and policies based on Mediterranean and Nordic diets are recommended or implemented in the WHO European Region, and is there evidence of effectiveness in reducing noncommunicable diseases? Copenhagen; 2018. 30091868

[96] Kibret KT, Chojenta C, Gresham E, Tegegne TK, Loxton D (2019). Maternal dietary patterns and risk of adverse pregnancy (hypertensive disorders of pregnancy and gestational diabetes mellitus) and birth (preterm birth and low birth weight) outcomes: a systematic review and meta-analysis. Public Health Nutr.

[97] Parlapani E, Agakidis C, Karagiozoglou-Lampoudi T, Sarafidis K, Agakidou E, Athanasiadis A (2019). The Mediterranean diet adherence by pregnant women delivering prematurely: association with size at birth and complications of prematurity. J Matern Neonatal Med.

[98] Timmermans S, Steegers-Theunissen RPM, Vujkovic M, Bakker R, Den Breeijen H, Raat H (2011). Major dietary patterns and blood pressure patterns during pregnancy: the Generation R Study. Am J Obstet Gynecol.

[99] Schoenaker DAJM, Soedamah-Muthu SS, Callaway LK, Mishra GD (2015). Prepregnancy dietary patterns and risk of developing hypertensive disorders of pregnancy: results from the Australian Longitudinal Study on Women’s Health. Am J Clin Nutr.

[100] Hillesund ER, Øverby NC, Engel SM, Klungsøyr K, Harmon QE, Haugen M (2014). Associations of adherence to the New Nordic Diet with risk of preeclampsia and preterm delivery in the Norwegian Mother and Child Cohort Study (MoBa). Eur J Epidemiol.

[101] Arvizu M, Bjerregaard AA, Madsen MT, Granström C, Halldorsson TI, Olsen SF (2020). Sodium intake during pregnancy, but not other diet recommendations aimed at preventing cardiovascular disease, is positively related to risk of hypertensive disorders of pregnancy. J Nutr.

[102] Fulay AP, Rifas-Shiman SL, Oken E, Perng W (2018). Associations of the dietary approaches to stop hypertension (DASH) diet with pregnancy complications in Project Viva. Eur J Clin Nutr.

[103] Borgen I, Aamodt G, Harsem N, Haugen M, Meltzer HM, Brantsæter AL (2012). Maternal sugar consumption and risk of preeclampsia in nulliparous Norwegian women. Eur J Clin Nutr.

[104] Belizan JM, Villar J (1980). The relationship between calcium intake and edema-, proteinuria-, and hypertension-gestosis: an hypothesis. Am J Clin Nutr.

[105] Sukonpan K, Phupong V (2005). Serum calcium and serum magnesium in normal and preeclamptic pregnancy. Arch Gynecol Obstet.

[106] Belizán JM, Villar J, Repke J (1988). The relationship between calcium intake and pregnancy-induced hypertension: up-to-date evidence. Am J Obstet Gynecol.

[107] Cormick G, Belizán JM (2019). Calcium intake and health. Nutrients.

[108] Khan M, Sharma S. Physiology, parathyroid hormone (PTH). StatPearls. StatPearls Publishing; 2018. http://www.ncbi.nlm.nih.gov/pubmed/29763115. Accessed 30 May 2020. 29763115

[109] Kawashima H (1990). Parathyroid hormone causes a transient rise in intracellular ionized calcium in vascular smooth muscle cells. Biochem Biophys Res Commun.

[110] Okoror CEM, Enabudoso EJ, Okoror OT, Okonkwo CA (2020). Serum calcium-magnesium ratio in women with pre-eclampsia at a tertiary hospital in Nigeria. Int J Gynecol Obstet.

[111] Belizán J, Villar J, Self S, Pineda O, González I, Sainz E (1984). The mediating role of the parathyroid gland in the effect of low calcium intake on blood pressure in the rat. Arch Latinoam Nutr.

[112] Villa-Etchegoyen C, Lombarte M, Matamoros N, Belizán JM, Cormick G, Villa-Etchegoyen C (2019). Mechanisms involved in the relationship between low calcium intake and high blood pressure. Nutrients.

[113] de Brito Galvao JF, Nagode LA, Schenck PA, Chew DJ (2013). Calcitriol, calcidiol, parathyroid hormone, and fibroblast growth factor-23 interactions in chronic kidney disease. J Vet Emerg Crit Care.

[114] Gupta A, Kant S, Pandav CS, Gupta SK, Rai SK, Misra P (2016). Dietary calcium intake, serum calcium level, and their association with preeclampsia in rural North India. Indian J Community Med.

[115] Kant S, Haldar P, Gupta A, Lohiya A (2019). Serum calcium level among pregnant women and its association with pre-eclampsia and delivery outcomes: a cross-sectional study from North India. Nepal J Epidemiol.

[116] Elmugabil A, Hamdan HZ, Elsheikh AE, Rayis DA, Adam I, Gasim GI (2016). Serum calcium, magnesium, zinc and copper levels in Sudanese women with preeclampsia. PLoS ONE.

[117] Owusu Darkwa E, Antwi-Boasiako C, Djagbletey R, Owoo C, Obed S, Sottie D (2017). Serum magnesium and calcium in preeclampsia: a comparative study at the Korle-Bu Teaching Hospital, Ghana. Integr Blood Press Control.

[118] Ephraim RKD, Osakunor DNM, Denkyira SW, Eshun H, Amoah S, Anto EO (2014). Serum calcium and magnesium levels in women presenting with pre-eclampsia and pregnancy-induced hypertension: a case-control study in the Cape Coast metropolis, Ghana. BMC Pregnancy Childbirth.

[119] Abdellah AAA, Abdrabo AA (2014). Assessment of serum calcium, magnesium, copper and zinc levels in Sudanese pregnant women with pre-eclampsia. Glob Adv Res J Med Med Sci.

[120] Guo X, Xu L, Huang J, Zhao M. Case-control study on serum calcium and magnesium levels in women presenting with preeclampsia. Chronic Dis Prev Rev. 2017. www.cancercellresearch.org. Accessed 16 May 2020.

[121] Kanagal DV, Rajesh A, Rao K, Devi UH, Shetty H, Kumari S (2014). Levels of serum calcium and magnesium in pre-eclamptic and normal pregnancy: a study from coastal India. J Clin Diagn Res.

[122] Tavana Z, Hosseinmirzaei S (2013). Comparison of maternal serum magnesium level in pre-eclampsia and normal pregnant women. Iran Red Crescent Med J.

[123] Vafaei H, Dalili M, Hashemi SA (2015). Serum concentration of calcium, magnesium and zinc in normotensive versus preeclampsia pregnant women: a descriptive study in women of Kerman province of Iran. Iran J Reprod Med.

[124] Kinshella M-LW, Omar S, Scherbinsky K, Vidler M, Magee LA, von Dadelszen P (2021). Effects of maternal nutritional supplements and dietary interventions on placental complications: an umbrella review, meta-analysis and evidence map. Nutrients.

[125] Mori R, Ota E, Middleton P, Tobe-Gai R, Mahomed K, Bhutta ZA. Zinc supplementation for improving pregnancy and infant outcome. Cochrane Database Syst Rev. 2012;(7):CD000230. 10.1002/14651858.CD000230.pub4 22786472

[126] Rumbold A, Ota E, Nagata C, Shahrook S, Crowther CA. Vitamin C supplementation in pregnancy. Cochrane Database Syst Rev. 2015;(9):CD004072. 10.1002/14651858.CD004072.pub3 PMC9039972 26415762

[127] Pena-Rosas JP, De-Regil LM, Garcia-Casal MN, Dowswell T, PenaRosas PJ, DeRegil ML, et al. Daily oral iron supplementation during pregnancy. Cochrane Database Syst Rev. 2015;(7):CD004736. 10.1002/14651858.CD004736.pub5 PMC8918165 26198451

[128] Ramakrishnan U, Grant F, Goldenberg T, Zongrone A, Martorell R (2012). Effect of women’s nutrition before and during early pregnancy on maternal and infant outcomes: a systematic review. Paediatr Perinat Epidemiol.

[129] Fowles ER, Walker LO, Marti CN, Ruiz RJ, Wommack J, Bryant M (2012). Relationships among maternal nutrient intake and placental biomarkers during the 1st trimester in low-income women. Arch Gynecol Obs.

[130] Reijnders IF, Mulders AGMGJ, Van Der Windt M, Steegers EAP, Steegers-Theunissen RDSPM (2019). The impact of periconceptional maternal lifestyle on clinical features and biomarkers of placental development and function: a systematic review. Hum Reprod Update.

