# Supplementary material for: Maternal nutritional risk factors for pre-eclampsia incidence: findings from a narrative scoping review
Source: Reprod Health. 2022 Sep 5;19:188. doi: 10.1186/s12978-022-01485-9 (PMC9442926; doi:10.1186/s12978-022-01485-9)
Supplement: Supplementary file 2 — Additional file 2. Search terms. [file 12978_2022_1485_MOESM2_ESM.docx]

**Additional File 2: Search terms**

**Search strategy for Medline Ovid**

| 1. Nutrit* supplement or vitamin* or mineral* or micronutrit* |
| --- |
| 1. (Minerals/ or Micronutrients/ or Vitamins/ or exp Diet/) and Humans/ |
| 1. (Dietary Proteins/ or Potassium, Dietary/ or Dietary Exposure/ or Dietary Sucrose/ or Dietary Carbohydrates/ or Sodium, Dietary/ or Dietary Sugars/ or Dietary Fats/ or Cholesterol, Dietary/ or Calcium, Dietary/ or Plant Proteins, Dietary/ or Sodium Chloride, Dietary/ or Dietary Fiber/ or Fatty Acids/) and Humans/ |
| 1. Diet* or diet* qualit* or health* diet* or diet* intervention* or Mediterranean diet* or Mediterranean style diet* or Mediterranean type diet* or Dietary approaches to stop hypertension or DASH diet* |
| 1. Diet, Fat-restricted/ or Diet, High-protein/ or diet, Mediterranean/ or Diet, Reducing/ or Diet, Sodium-restricted/ or Diet, Vegetarian/ or Dietary Approaches to Stop Hypertension/ or Healthy diet/ |
| 1. Diet* pattern* or Eat* pattern* or Diet* habit or Diet* guideline* or Diet* adhere* or Diet* complian* or Nutrition* adhere* or Nutrition* complian* or Diet* score* or Diet* index* or Diet* indices |
| 1. 1 or 2 or 3 or 4 or 5 or 6 |
| 1. Pregnan* or Pregnan* complicat* or Placenta* pathology or Birth outcome* or Obstetric* outcome* |
| 1. Pregnancy/ or Pregnancy Complications/ or Pregnancy Outcome/ or Maternal Nutritional Physiological Phenomena/ or Prenatal Nutritional Physiological Phenomena/ |
| 1. Matern*mortality or Matern* death* or pregnancy death* or motherhood death* or women death* |
| 1. Maternal Mortality/ or Maternal Death/ |
| 1. Pregnan* hypertension or gestational hypertension or chronic hypertension or pre-eclampsia or preeclampsia or toxemia or eclampsia or HEELP syndrome |
| 1. Hypertension/ or Hypertension, Pregnancy-Induced/ or Pregnancy Complications, Cardiovascular/ or Pre-Eclampsia/ or HELLP Syndrome/ |
| 1. 8 or 9 or 10 or 11 or 12 or 13 |
| 1. 7 and 14 |
