# Supplementary material for: Maternal nutritional risk factors for pre-eclampsia incidence: findings from a narrative scoping review
Source: Reprod Health. 2022 Sep 5;19:188. doi: 10.1186/s12978-022-01485-9 (PMC9442926; doi:10.1186/s12978-022-01485-9)
Supplement: Supplementary file 3 — Additional file 3. PRISMA flow diagram. [file 12978_2022_1485_MOESM3_ESM.docx]

**Additional File 3: PRISMA flow diagram**

Records excluded
(n =4137)

Records screened
(n = 4369)

Records after duplicates removed
(n = 4369)

## Identification

## Eligibility

## Included

## Screening

Records identified through database searching
(n = 6572)

Full-text articles excluded
(n = 23)*

Full-text articles assessed for eligibility
(n = 52)

Articles included from search stage 2 (expanded search)
(n = 29)

Articles included from search stage 1 (systematic search)
(n = 29)

Articles included in qualitative synthesis
(n = 58)

**see table below for excluded articles*

**Excluded studies**

| **Reference** | **Article title** | **Reason for exclusion** |
| --- | --- | --- |
| Abu-Saad and Fraser 2010 | Maternal nutrition and birth outcomes. | Did not cover outcome of interest (pre-eclampsia) |
| Attree 2005 | Low-income mothers, nutrition and health: a systematic review of qualitative evidence | Did not cover outcome of interest (pre-eclampsia) |
| Bulloch et al 2018 | Maternal folic acid supplementation for the prevention of preeclampsia: A systematic review and meta-analysis | Wrong study design (supplementation or fortification studies) |
| Chia et al 2019 | Maternal Dietary Patterns and Birth Outcomes: A Systematic Review and Meta-Analysis. | Did not cover outcome of interest (pre-eclampsia) |
| Doyle et al 2017 | Determinants of dietary patterns and diet quality during pregnancy: a systematic review with narrative synthesis | Did not cover outcome of interest (pre-eclampsia) |
| Drueke 1999 | Dietary calcium and blood pressure | Wrong population (not in pregnancy) |
| Fang et al 2021 | Maternal vitamin D deficiency during pregnancy and low birth weight: a systematic review and meta-analysis | Did not cover outcome of interest (pre-eclampsia) |
| Harika et al 2017 | Micronutrient Status and Dietary Intake of Iron, Vitamin A, Iodine, Folate and Zinc in Women of Reproductive Age and Pregnant Women in Ethiopia, Kenya, Nigeria and South Africa: A Systematic Review of Data from 2005 to 2015 | Did not cover outcome of interest (pre-eclampsia) |
| Hu et al 2018 | Maternal Vitamin D Deficiency and the Risk of Small for Gestational Age: A Meta-analysis | Did not cover outcome of interest (pre-eclampsia) |
| Kjollesdal and Holmboe-Ottesen 2014 | Dietary Patterns and Birth Weight-a Review | Did not cover outcome of interest (pre-eclampsia) |
| Lewis et al 1998 | Drug and environmental factors associated with adverse pregnancy outcomes. Part III: Folic acid: pharmacology, therapeutic recommendations, and economics | Did not cover outcome of interest (pre-eclampsia); Wrong study design (supplementation or fortification studies) |
| Mantovani et al 2014 | Folic acid supplementation and preterm birth: results from observational studies | Did not cover outcome of interest (pre-eclampsia); Wrong study design (supplementation or fortification studies) |
| Murphy et al 2014 | Associations of consumption of fruits and vegetables during pregnancy with infant birth weight or small for gestational age births: a systematic review of the literature | Did not cover outcome of interest (pre-eclampsia) |
| Ponzetto et al 2019 | Prepregnancy calcium supplementation and pre-eclampsia | Wrong study design (supplementation or fortification studies) |
| Qin et al 2016 | Does Maternal Vitamin D Deficiency Increase the Risk of Preterm Birth: A Meta-Analysis of Observational Studies | Did not cover outcome of interest (pre-eclampsia) |
| Raghavan et al 2019 | Dietary patterns before and during pregnancy and birth outcomes: a systematic review | Did not cover outcome of interest (pre-eclampsia) |
| Rogne et al 2014 | Associations of Maternal Vitamin B12 Concentration in Pregnancy With the Risks of Preterm Birth and Low Birth Weight: A Systematic Review and Meta-Analysis of Individual Participant Data | Did not cover outcome of interest (pre-eclampsia) |
| Rogozinska et al 2014 | Variation in outcomes in trials reporting effects of diet and lifestyle based intervention on pregnancy outcomes: a systematic review. | Wrong study design (supplementation or fortification studies) |
| van Uitert et al 2013 | Influence of maternal folate status on human fetal growth parameters. | Did not cover outcome of interest (pre-eclampsia) |
| Wen and Walker 2005 | An exploration of health effects of folic acid in pregnancy beyond reducing neural tube defects | Did not cover outcome of interest (pre-eclampsia) |
| Yang et al 2016 | Periconceptional folic acid fortification for the risk of gestational hypertension and pre-eclampsia: a meta-analysis of prospective studies. | Wrong study design (supplementation or fortification studies) |
| Zerfu and Ayele 2013 | Micronutrients and pregnancy; effect of supplementation on pregnancy and pregnancy outcomes: a systematic review | Wrong study design (supplementation or fortification studies) |
| Zhang et al 2017 | Effect of folic acid supplementation on preterm delivery and small for gestational age births: A systematic review and meta-analysis | Did not cover outcome of interest (pre-eclampsia); Wrong study design (supplementation or fortification studies) |
